# Supplementary material for: Patient-derived tumoroids from CIC::DUX4 rearranged sarcoma identify MCL1 as a therapeutic target
Source: Nat Commun. 2025 Aug 21;16:7688. doi: 10.1038/s41467-025-62629-6 (PMC12370961; doi:10.1038/s41467-025-62629-6)

Supplementary Information

Supplementary Table 1. Clinical data of tumors used for organoid culture. Cases with successful model establishment are highlighted in green. ES, Ewing sarcoma, CDS, CIC::DUX4 sarcoma, S, surgery, R, radiotherapy, DOD, died of disease, NA, not available.

Case	Sex	Primary tumor site	Size	Stage	Metastasis site	Biopsy site	Biopsy timepoint
ES-ZH001	Female	Extremity (Clavicula)	<200ml	Metastatic	Bone, Lymf	from primary site	at Diagnosis
ES-ZH002	Female	Rip	<200ml	Localised		from primary site	at Diagnosis
ES-ZH004	Male	Pelvis	>200ml	Metastatic		from primary site	at Diagnosis
ES-ZH007	Male	Pelvis	>200ml	Metastatic	Bone, Lung	from metastatic site (Bone)	at Relapse
ES-ZH005	Female	Tibia	<200ml	Metastatic	Lung, Lymf	from metastatic site (Lung)	at time of progress
ES-ZH006	Male	Extremity (Tibia)	<200ml	Localised		from primary site	at Diagnosis
ES-ZH008	Male	Rip	<200ml	Localised		from primary site	at Diagnosis
ES-ZH009	Male	M. Ileacus	>200ml	Metastatic	Bone, Lung	from metastatic site (soft tissue)	at time of progress
ES-ZH010	Female	Extremity	<200ml	Localised		from primary site	at Diagnosis
ES-ZH014	Female	Extremity	<200ml	Localised		from primary site after induction chemo	at surgery after induction
ES-ZH011	Female	Parotis	<200ml	Localised		from primary site	at Diagnosis
ES-ZH012				Metastatic		from metastatic (Lung)	at Relapse
ES-ZH013	Male	Pelvis	<200ml	Metastatic	Bone, Lung	from primary site	at Diagnosis
ES-ZH016	Female	Intracranial		Localised		from primary site	at Diagnosis
CDS-ZH001	Female	Kidney		Metastatic (at relapse)		from primary site after induction chemo	at surgery after induction
CDS-ZH001-2	Female	Kidney		Metastatic (at relapse)		from metastatic (Lung)	at Relapse
CDS-ZH002	Female	Extremity (M. Gluteus)	<200ml	Metastatic (at relapse)	Lung	from metastatic (Lung)	at Relapse
CDS-ZH003	Female	Head&Neck	<200ml	Metastatic (at relapse)	Lung	from metastatic (Lung)	at Relapse
CDS-MUG CIDUS	Female	Pelvis		Metastatic	Lung	from primary site	at surgery after induction

Case	Local treatment	Histologic response	Systemic treatment protocol	Outcome
ES-ZH001	S+R	Good	Euro Ewing 2008	DOD
ES-ZH002	S	Good	Euro Ewing 2008	Alive
ES-ZH004	R	NA	Euro Ewing 2008	DOD
ES-ZH007	R	NA	rEECur	DOD
ES-ZH005	S+R	Unknown	Euro Ewing 2008	DOD
ES-ZH006	S+R	NA (RT pre-OP)	Euro Ewing 2008	Alive
ES-ZH008	S+R	Poor	Euro Ewing 2012	Alive
ES-ZH009	R	NA	Euro Ewing 2012	DOD
ES-ZH010	S	NA	Euro Ewing 2012	Alive
ES-ZH014	S	Good	Euro Ewing 2012	Alive
ES-ZH011	R	NA	Euro Ewing 2012	Alive
ES-ZH012				
ES-ZH013	R	NA	Euro Ewing 2012	Alive
ES-ZH016	S+R	NA	Euro Ewing 2012	Alive
CDS-ZH001	S+R	Poor	Euro Ewing 2012	DOD
CDS-ZH001-2		NA	rEECur	DOD
CDS-ZH002	S+R		Euro Ewing /rEECur	DOD
CDS-ZH003		Poor		DOD
CDS-MUG CIDUS	S+R		VCR, Ifosfamide, Adriamycin, Actinomycin	DOD

Supplementary Table 2. Sarcoma classifier scores for CDS and EwS models and cell lines.

Model	Score Sarcoma classifier
CDS-ZH001	0.96155
CDS-ZH002	0.9786
CDS-ZH003	0.99718
ES-ZH001	0.99994
ES-ZH002	0.87981
ES-ZH004	0.99963
ES-ZH007	0.98036
ES-ZH008	0.99996
ES-ZH009	0.66323
SKNMC	0.06103
A673	0.67695
RDES	0.99855
TC71	0.40918

Supplementary Table 3. Pathways associated with genes differentially expressed between CDS and EwS. Normalized enrichment scores (NES), number of genes and adjusted p-values are depicted for individual pathways.

Direction	GSEA analysis: CDS vs ES Pathways	NES	Genes	adj.Pval
Down	ZHANG TARGETS OF EWSR1 FLI1 FUSION	-0.7225	82	1.70E-06
	RUNNE GENDER EFFECT UP	-0.9765	9	6.10E-06
	STAEGE EWING FAMILY TUMOR	-0.8379	31	3.70E-05
	PYEON CANCER HEAD AND NECK VS CERVICAL DN	-0.7853	17	5.60E-02
	WP BILE ACIDS SYNTHESIS AND ENTEROHEPATIC CIRCULATION	-0.9322	5	7.20E-02
	WEBER METHYLATED HCP IN FIBROBLAST DN	-0.7623	17	8.50E-02
	ZAIDI OSTEOBLAST TRANSCRIPTION FACTORS	-0.7881	14	9.30E-02
Up	WP HYPOTHESIZED PATHWAYS IN PATHOGENESIS OF CARDIOVASCU	0.7359	24	3.20E-02
	SOUCEK MYC TARGETS	0.9236	6	4.40E-02
	CHUANG OXIDATIVE STRESS RESPONSE UP	0.7478	20	4.40E-02
	MAHADEVAN GIST MORPHOLOGICAL SWITCH	0.8515	10	4.70E-02
	KOBAYASHI EGFR SIGNALING 6HR DN	0.7854	15	5.20E-02
	NIKOLSKY BREAST CANCER 14Q22 AMPLICON	0.8058	12	7.00E-02
	JEON SMAD6 TARGETS UP	0.7173	20	7.40E-02
	DORSEY GAB2 TARGETS	0.7159	23	7.40E-02
	LU TUMOR ENDOTHELIAL MARKERS UP	0.7165	20	7.60E-02
	BOYLAN MULTIPLE MYELOMA PCA1 DN	0.8539	8	8.10E-02
	CLASPER LYMPHATIC VESSELS DURING METASTASIS UP	0.747	18	8.10E-02
	DASU IL6 SIGNALING SCAR DN	0.7689	13	9.30E-02
	KORKOLA EMBRYONIC CARCINOMA VS SEMINOMA UP	0.7526	15	9.30E-02
**Direction**	**GSEA analysis: CDS vs ES Pathways**	**NES**	**Genes**	**adj.Pval**
Down	Nicotine addiction	-0.6717	30	0.086
Up	PI3K-Akt signaling pathway	0.4826	257	0.00011
	Platelet activation	0.562	100	0.0032
	Relaxin signaling pathway	0.5444	102	0.0051
	Notch signaling pathway	0.645	51	0.013
	Cytokine-cytokine receptor interaction	0.4842	131	0.019
	ECM-receptor interaction	0.5821	62	0.027
	AGE-RAGE signaling pathway in diabetic complications	0.5338	87	0.027
	Tuberculosis	0.4789	124	0.035
	Focal adhesion	0.4462	167	0.039
	Endocrine resistance	0.51	83	0.06
	Chronic myeloid leukemia	0.5306	69	0.074
	Neurotrophin signaling pathway	0.4709	106	0.074
	Apelin signaling pathway	0.4694	104	0.074
	Rheumatoid arthritis	0.5663	51	0.088
	Glycosaminoglycan biosynthesis-chondroitin sulfate/dermatan sulfate	0.7405	16	0.099
	Malaria	0.6374	29	0.099
	Phagosome	0.459	108	0.099
	Lysosome	0.4492	114	0.099
	Insulin signaling pathway	0.4459	118	0.099

Supplementary Table 4. Composition of drug library.

Drug	Drug	Drug
10058F4	Entospletinib	Oxaliplatin
6H05	Entrectinib	Paclitaxel
A-1210477	Epacadostat	Palbociclib
A-485	EPZ011989	Panobinostat
Abemaciclib	Erdafitinib	Pazopanib
Abexinostat	Erlotinib	Pevonedistat
Acalabrutinib	Etoposide	Pexidartinib
Adavosertib	Everolimus	PF3758309
AEE788	Fedratinib	PF4708671
AEW541	Fimepinostat	PF477736
Afatinib	Fludarabine	PF562271
Afuresertib	GDC0152	Pimozide
Alectinib	Gemcitabine	Pomalidomide
Alisertib	Gemtuzumab	Ponatinib
Alpelisib	Gilteritinib	PQR514
Alvocidib	Givinostat	PQR620
Amsacrine	Glasdegib	Prednisone
Apabetalone	GSK269962A	PRN1371
AS-1842856	GSK690693	Quizartinib
AT9283	GW842166X	Rabusertib
Axitinib	Homoharringtonine	RAF-265
AZ20	HSP-990	Ralimetinib
Azacitidine	Hydroperoxycyclophosphamide	Ravoxertinib
AZD1080	Hydroperoxyifosfamide	Regorafenib
AZD1208	I-BRD9	Regorafenib Monohydrate
AZD7762	Ibrutinib	Repotrectinib
AZD8055	Idarubicin	Resveratrol
Barasertib	Idasanutlin	RG2833
BHG712	Idelalisib	Ribociclib
BI-2536	Ifosfamide	RKI1447
BI-D1870	IKK16	Rucaparib
Birabresib	Imatinib	Ruxolitinib
Birinapant	Infigratinib	S-63845
BIX01294	IOX2	Samotolisib
BMS345541	IPA3	Saracatinib
Bortezomib	Ipatasertib	SB216763
Bosutinib	Irinotecan	Selinexor
Brigatinib	Ivermectin	Selisistat
BSK805	Ivosidenib	Selumetinib
Buparlisib	Ixazomib	SGI1776
Busulfan	JIB04	Sirolimus
BX-795	JQ1	SN-38
Cabozantinib	KU55933	Sonidegib
Carboplatin	KW-2478	Sorafenib
Carfilzomib	Lapatinib	Sotorasib
Carmustine	Larotrectinib	Spebrutinib
Ceralasertib	LCL161	Sunitinib
Ceritinib	Lenalidomide	T0070907
Chloroquine	Lenvatinib	Talazoparib
Clofarabine	Linifanib	Tazemetostat
Cobimetinib	Linsitinib	Temozolomide
CPI169	Lorlatinib	Temsirolimus
Crenigacestat	Luminespib	Tepotinib
Crenolanib	Melphalan	Thioguanine
Crizotinib	Mercaptopurine	Thiotepa
Cyclophosphamide	Methotrexate	THZ1
Cyclosporin A	Midostaurin	Tirbanibulin
Cytarabine	Mirdametinib	Tivantinib
Dabrafenib	Mitomycin	TK216
Dacarbazine	Mitoxantrone	Tofacitinib
Dactinomycin	MK-2206	Topotecan
Dactolisib	MK-8776	Torin-1
DAPT	Mocetinostat	Torkinib
Dasatinib	Molibresib	Trametinib
Daunorubicin	Momelotinib	Uprosertib
Decitabine	Motolimod	Valproic acid
Degrasyn	Moxidectin	Veliparib
Delanzomib	MRTX849	Vemurafenib
Dexamethasone	Navitoclax	Venetoclax
Dibenzazepine	Necrostatin-2	Verdinexor
Dinaciclib	Neflamapimod	Vinblastine
Docetaxel	Nelarabine	Vincristine
Dovitinib	Nexturastat A	Vinorelbine
Doxorubicin	Nilotinib	Vismodegib
Duvelisib	NSC23766	Volasertib
Elesclomol	NU7441	Vorinostat
Elimusertib	Nutlin-3	XAV-939
Eltanexor	Obatoclax	YK-4-279
Enasidenib	Olaparib	YM155
Ensartinib	Omipalisib	Zelavespib
Entinostat	Onalespib	ZM-447439

Supplementary Table 5. IC50 values of S63845 measured with 45 sarcomas from indicated entities.

Cells	Sarcoma entity	IC50 / nM
CDS-ZH001	CIC-DUX4 sarcoma	6.352
CDS-ZH003	CIC-DUX4 sarcoma	8.158
CDS-ZH001-2	CIC-DUX4 sarcoma	10.31
ES-ZH007	Ewing sarcoma	24.53
CDS-ZH002	CIC-DUX4 sarcoma	44.3
DDLS-ZH002	Dedifferentiated liposarcoma	93.6
ES-ZH004	Ewing sarcoma	187.3
ES-ZH009	Ewing sarcoma	207.8
SS-ZH003	Synovial Sarcoma	224.4
ES-BE002	Ewing sarcoma	366.5
RT-ZH001	Rhabdoid Tumor	397.6
RMS-ZH010-2	Rhabdomyosarcoma	438.7
ES-ZH008	Ewing sarcoma	449.7
ES-ZH016	Ewing sarcoma	508.5
RMS-444	Rhabdomyosarcoma	661.7
RMS-Wi001	Rhabdomyosarcoma	1029
UPS-ZH003	Undifferentiated pleomorphic sarcoma	1108
RMS Berlin 13870	Rhabdomyosarcoma	1594
RMS-410	Rhabdomyosarcoma	1894
RMS-ZH009	Rhabdomyosarcoma	1950
RMS Berlin 10752	Rhabdomyosarcoma	2095
RMS-ZH003	Rhabdomyosarcoma	2873
OS-ZH009	Osteosarcoma	3169
RMS-ZH010	Rhabdomyosarcoma	3678
RMS Berlin 13454	Rhabdomyosarcoma	3840
RMS Berlin 11492	Rhabdomyosarcoma	3992
RMS-ZH016	Rhabdomyosarcoma	4039
RMS-ZH017	Rhabdomyosarcoma	4121
RMS-ZH018	Rhabdomyosarcoma	5580
RMS Berlin 13304	Rhabdomyosarcoma	6454
RMS-127	Rhabdomyosarcoma	8976
SJRHB013759_X1	Rhabdomyosarcoma	9134
RMS IC-pPDX-104	Rhabdomyosarcoma	11976
RMS Berlin 12181	Rhabdomyosarcoma	12540
ES-ZH001	Ewing sarcoma	12732
RMS-ZH004-3	Rhabdomyosarcoma	17592
RMS-ZH014	Rhabdomyosarcoma	17762
OS-BE006	Osteosarcoma	21135
ALT-ZH001	Atypical lipomatous tumor	31768
RMS Berlin 13933	Rhabdomyosarcoma	36284
OS-BE005	Osteosarcoma	42162
GCT-ZH001	Giant Cell Tumor of bone	54119
RMS Berlin 14419	Rhabdomyosarcoma	71271
SS-ZH005	Synovial Sarcoma	110654
UBS-ZH001	Undifferentiated pleomorphic sarcoma	368604

Supplementary Table 6. Small molecule screening data.

Category	Parameter	Description
Assay	Type of assay	In vitro cell based assay
	Target	Cell viability
	Primary measurement	Detection of ATP levels
	Key reagents	Cell Titer Glo (Promega)
	Assay protocol	https://ch.promega.com/-/media/files/resources/protocols/technical-bulletins/0/celltiter-glo-luminescent-cell-viability-assay-protocol.pdf?rev=30e8ec640fdd4866b207e28c0cbc497c&sc_lang=en
	Additional comments	
Library	Library size	245 drugs
	Library composition	Broad range of chemotherapeutics used for sarcoma treatment plus experimental drugs directed against a broad range of signaling pathways and potential targets for cancer therapy.
	Source	Selleckchem
	Additional comments	
Screen	Format	384-well plates (Greiner Bio-One, μ-clear No. 781098)
	Concentration(s) tested	10, 100, 1000, 10000 nM. 0.2% DMSO
	Plate controls	DMSO only controls
	Reagent/ compound dispensing system	Echo 650 liquid handler
	Detection instrument and software	BioTek Cytation3 Imaging Reader and the BioTek Gen5 2.07.17 software
	Assay validation/QC	Negative control coefficient of variation below 0.18
	Correction factors	None
	Normalization	Cell viability was normalized to DMSO controls
	Additional comments	
Post-HTS analysis	Hit criteria	Top differential drug sensitivity score (dDSS) when compared to a cohort of sarcoma
	Hit rate	<5
	Additional assay(s)	Retesting of initial hits in original assay, cell death assays (flow cytometry, high content imaging), xenograft assay in mice.
	Confirmation of hit purity and structure	No
	Additional comments	Validation with an additional compound with a different structure directed against the same target.

Extended legend for Figure 2a

Abbreviations: LIPO, lipoma; MLS, myxoid liposarcoma; WDLS/DDLS, well differentiated liposarcoma/dedifferentiated liposarcoma; NFA, nodular fasciitis; MO, myositis ossificans; MP, myositis proliferans; DTFM, desmoid-type fibromatosis; DFSP, dermatofibrosarcoma protuberans; SFT, solitary fibrous tumor; IMT, inflammatory myofibroblastic tumor; IFS, infantile fibrosarcoma; LGFMS, low-grade fibromyxoid sarcoma; SEF, sclerosing epithelioid fibrosarcoma; LMO, leiomyoma; LMS, leiomyosarcoma; RMS (EMB), embryonal rhabdomyosarcoma; RMS (ALV), alveolar rhabdomyosarcoma; RMS (MYOD1), rhabdomyosarcoma with MYOD1 mutation; ALMO/MPC, angioleiomyoma/myopericytoma; EHE, epithelioid hemangioendothelioma; AS, angiosarcoma; GIST, gastrointestinal stromal tumor; SWN, schwannoma; NFB, neurofibroma; NFB (PLEX), plexiform neurofibroma; MPNST, malignant peripheral nerve sheath tumor; AFX/PDS, atypical fibroxanthoma/pleomorphic dermal sarcoma; AFH, angiomatoid fibrous histiocytoma; OFMT, ossifying fibromyxoid tumor; SYSA, synovial sarcoma; ES, epithelioid sarcoma; ASPS, alveolar soft part sarcoma; CCS, clear cell sarcoma of soft parts; EMCS, extraskeletal myxoid chondrosarcoma; DSRCT, desmoplastic small round cell tumor; MRT, malignant rhabdoid tumor; USARC, undifferentiated sarcoma; CCSK, clear cell sarcoma of the kidney; ESS (LG), low-grade endometrial stromal sarcoma; ESS (HG), high-grade endometrial stromal sarcoma; SCC (CUT), cutaneous squamous cell carcinoma; MEL (CUT), cutaneous melanoma; SARC, sarcoma; CTRL, control; MUS, muscle tissue; REA, reactive tissue; CB, chondroblastoma; CSA, chondrosarcoma; CSA (MES), mesenchymal chondrosarcoma; CSA (CC), clear cell chondrosarcoma; OB, osteoblastoma; OS (HG), high-grade conventional osteosarcoma; SBRCT, small blue round cell tumor; GCTB, giant cell tumor of bone; CHORD, chordoma; DD, dedifferentiated; FDY, fibrous dysplasia; LCH, Langerhans cell histiocytosis.

Supplementary Figure S1. In vitro culture of EwS and CDS models.

a

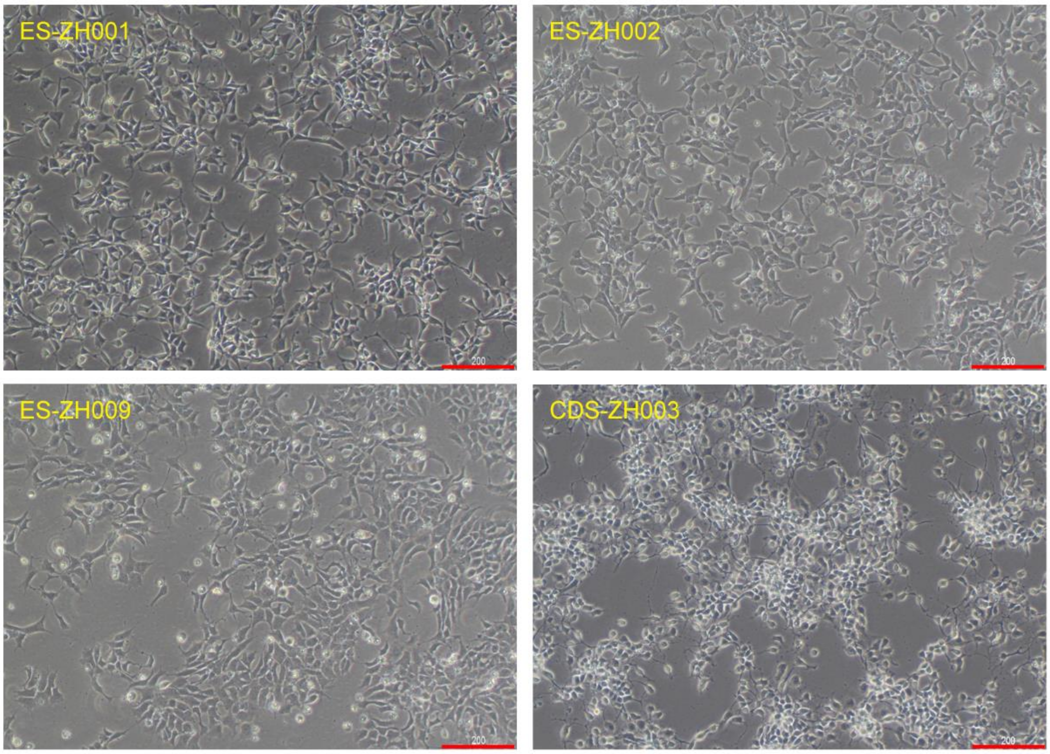

Supplementary Figure S1. Phase-contrast microscopy pictures illustrating the morphology of indicated EwS and CDS models grown as 2D monolayers. Scale bar, 200 μm.

Supplementary Figure S2. Effect of growth factors on viability of EwS and CDS cells.

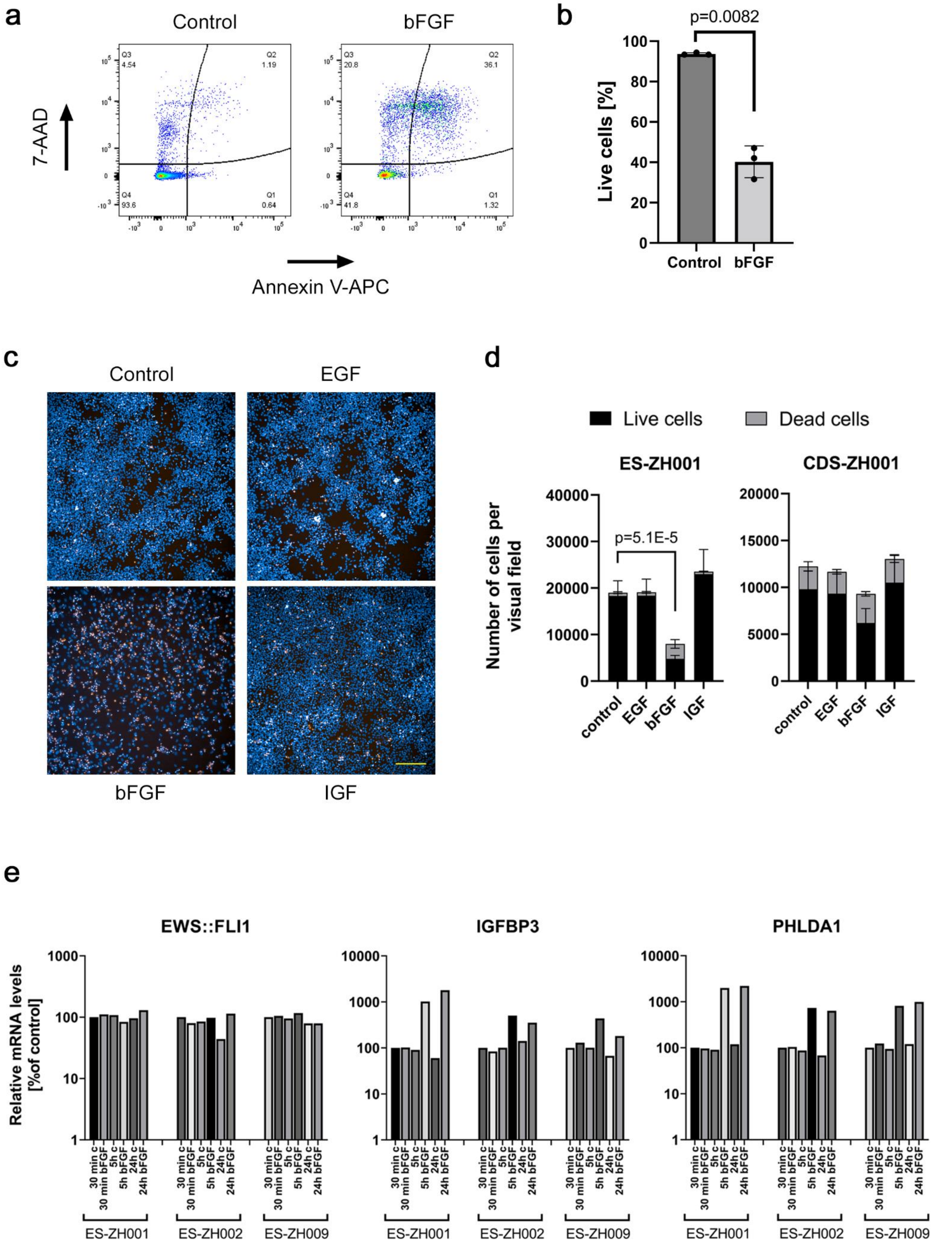

Supplementary Figure S2. **a**, Detection of death of ES-ZH001 cells after treatment with 10 ng/ml bFGF for 3 days by Annexin V and 7-AAD staining followed by flow cytometric analysis. Representative pseudocolor density plots illustrate

the number of live and dead cells. Plots are representative for n=3 independent experiments. **b**, Percentage of live cells, defined as Annexin V-negative and 7-AAD-negative, in the experiment described in a. n=3 independent experiments, two-tailed paired t-test. **c**, Fluorescence microscopy images of ES-ZH001 cells after treatment with indicated growth factors for 4 days. Cells were stained with Hoechst and propidium iodide to stain all cells and dead cells, respectively. Images were acquired using an Operetta high-content analysis system. Scale bar, 200 μm. Images are representative for n=3 independent experiments. **d**, Quantification of live and dead cells by image-based analysis. ES-ZH001 (upper panel) and CDS-ZH001 (lower panel) cells were treated with indicated growth factors for 4 or 7 days, respectively. Live and dead cells were identified based on images as shown in c. ES-ZH001, n=3 independent experiments, two-way ANOVA, Šídák's multiple comparisons test, the depicted p-value refers to live cells. CDS-ZH001, n=2 independent experiments. **e**, mRNA levels of indicated genes in three EwS tumoroid models after treatment with bFGF for different duration, as determined by qRT-PCR. n=1 independent experiment.

Supplementary Figure S3. STAG2 expression in EwS models.

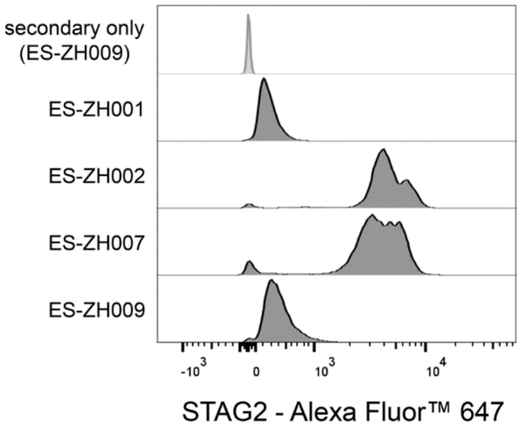

Supplementary Figure S3. STAG2 detection by flow cytometry. Indicated EwS-models were stained with an antibody against STAG2 and analysed by flow cytometry. n=1 independent experiment.

Supplementary Figure S4. S63845 IC50 values

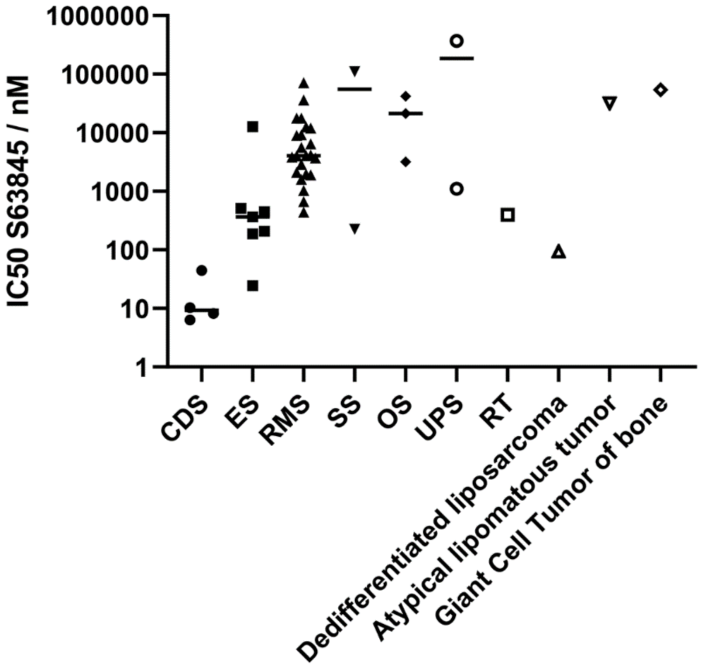

Supplementary Figure S4. IC50 values of S63845 for indicated sarcoma entities.

Supplementary Figure S5. Effect of BRD-810 on EwS and CDS models**.**

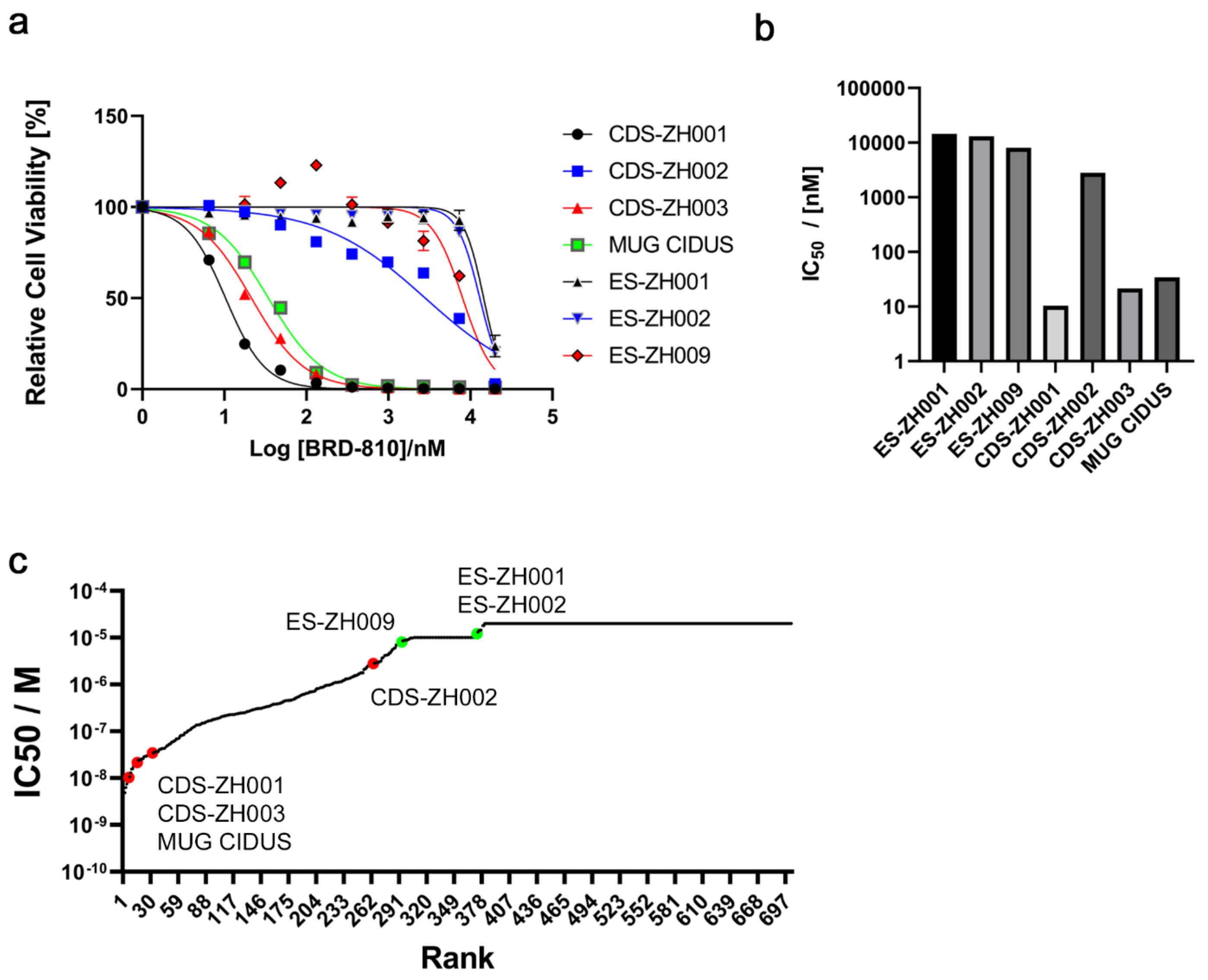

Supplementary Figure S5. **a**, Dose-response curves of the indicated CDS and EwS models treated with BRD-810 for 5 days. n=2 independent experiments. **b**, IC50 values of BRD-810 in the indicated CDS and EwS models. IC50 values were calculated from the data shown in a. **c**, Ranked IC50 values of BRD-810. IC50 values from CDS (red dots) and EwS (green dots) models were compared to a published dataset of IC50 values generated by PRISM analysis of 696 cell lines [1].

Supplementary Figure S6. Effect of MCL1 knockout on expression of BCL2 family proteins.

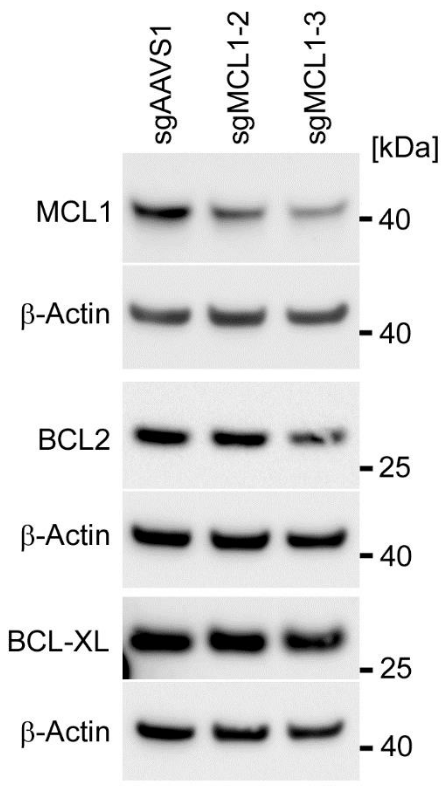

Supplementary Figure S6. Western blot analysis of the indicated proteins in lysates from CDS-ZH003 cells following MCL1 knockout by CRISPR. Cells were transduced with a construct expressing Cas9 and one of the indicated sgRNAs. Cell lysates were collected 3 days post-transduction. n=1 independent experiment.

Supplementary Figure S7. Expression of *MCL1* and *BCL2* in tumors and normal tissues.

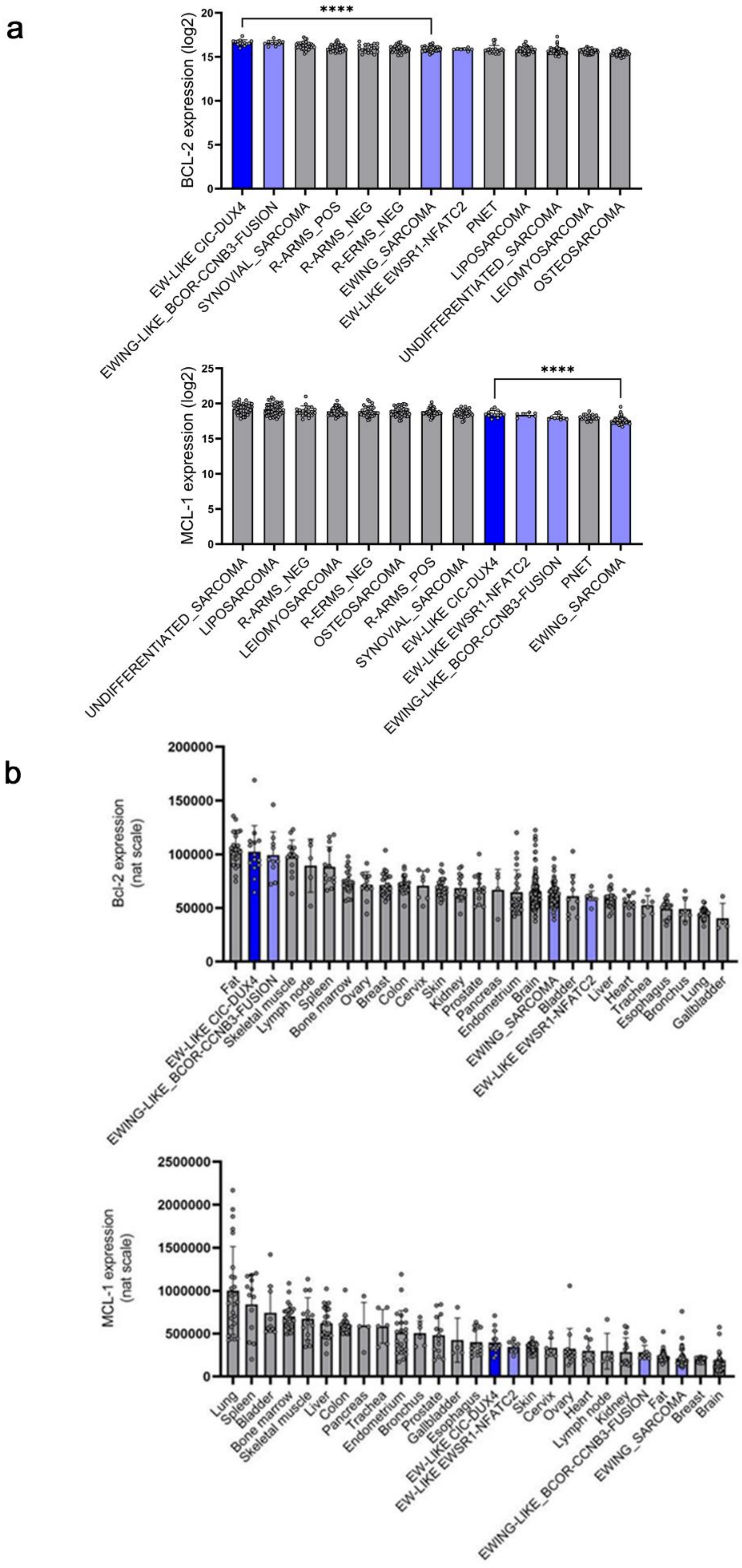

Supplementary Figure S7. **a** and **b**, Gene expression levels of *BCL2* (upper panels) and *MCL1* (lower panels) in different sarcoma (a) and various normal tissues (b) compared to EwS and CDS tumors. The data was generated using microarray-based gene expression analysis and was previously published [2].

Supplementary Figure S8. Binding of CIC::DUX4 to the *BCL2 and MCL1* loci.

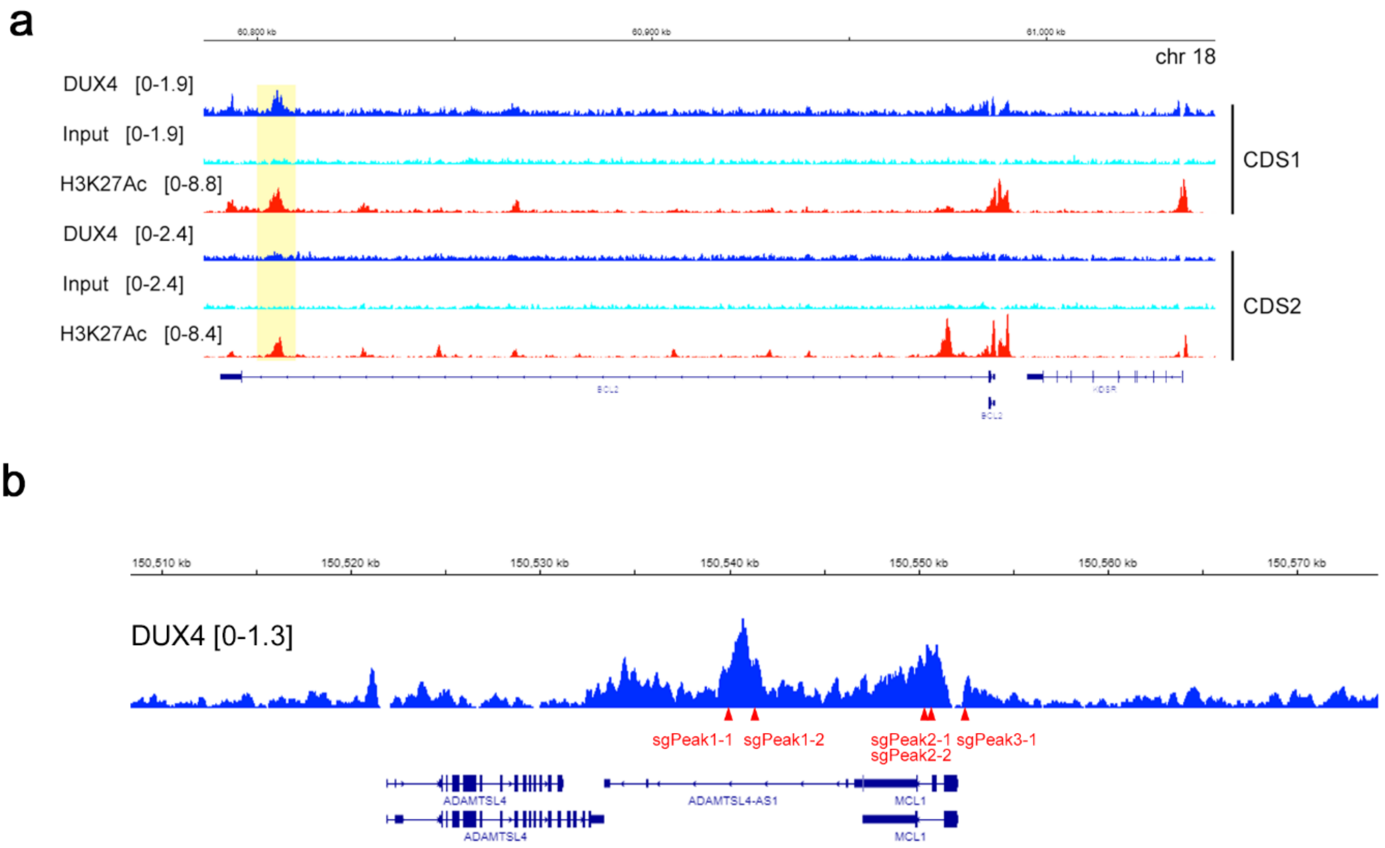

Supplementary Figure S8. **a**, ChIP-seq tracks for CIC::DUX4 and H3K27Ac at the *BCL2* locus in CDS1 and CDS2 cells. ChIP-seq data was previously published [3]. **b**, ChIP-seq tracks for CIC::DUX4 at the *MCL1* locus in CDS1 cells. The used ChIP-seq data was previously published [3]. Red arrowheads indicate the locations of the sgRNAs used for CRISPR interference.

Supplementary Figure S9. Effect of CIC::DUX4 silencing on viability of CDS cells.

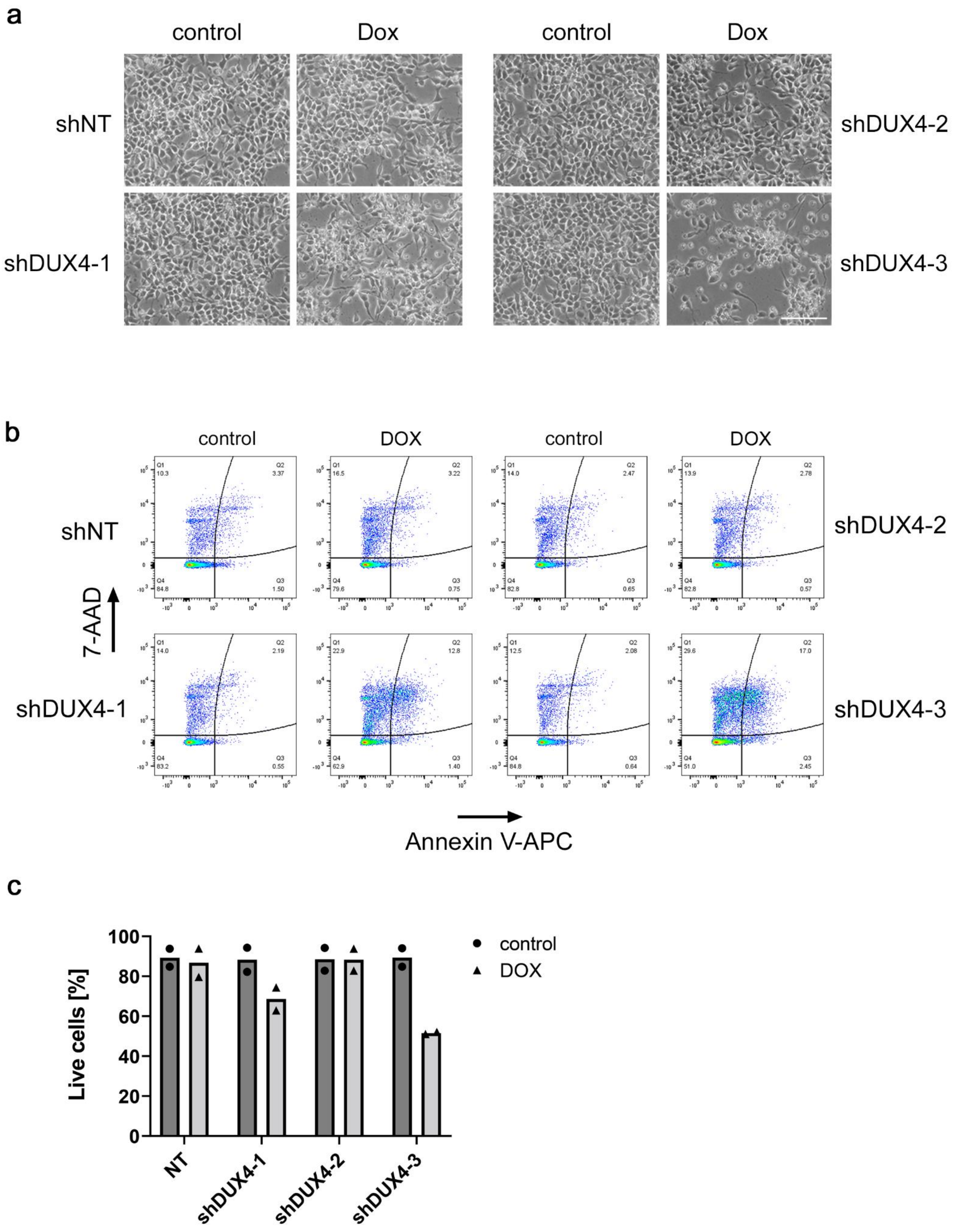

Supplementary Figure S9. **a**, Phase-contrast images of CDS-ZH003 cells transduced with indicated shRNA construct and treated with doxycycline for 4 days to induce shRNA expression or left untreated. Scale bar, 200 μm. Images are representative for n=3 independent experiments. **b**, Detection of cell death by flow cytometry of Annexin V and 7-AAD

stained cells. CDS-ZH003 cells containing the indicated doxycycline-inducible shRNA construct were treated with doxycycline for 4 days before analysis. Representative pseudocolor density plots illustrate the number of live and dead cells. Plots are representative for n=2 independent experiments. **c**, Quantification of live cells from flow cytometry analyses as shown in b. Live cells were defined as Annexin V- and 7-AAD-negative. n=2 independent experiments.

Supplementary Figure S10. Detection of CIC and CIC::DUX4 isoforms expressed in CDS cells**.**

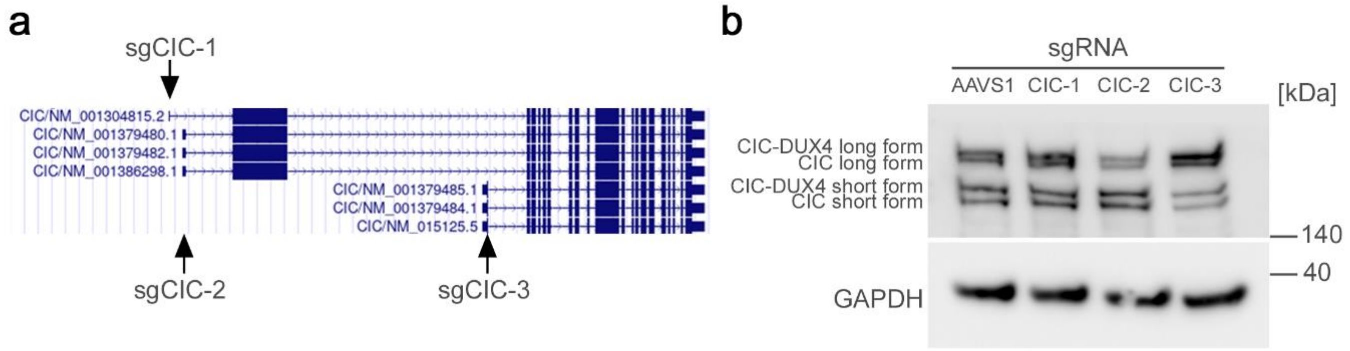

Supplementary Figure S10. **a**, Known transcripts of *CIC* with location of the sgRNAs used for CRISPR interference. **b**, Western blot detection of CIC and CIC::DUX4 after CRISPR interference with indicated sgRNAs in CDS-ZH003 cells. n=1 independent experiment.

Supplementary Figure S11. Effect of CIC::DUX4 silencing on *GAPDH* and *B2M* transcript levels.

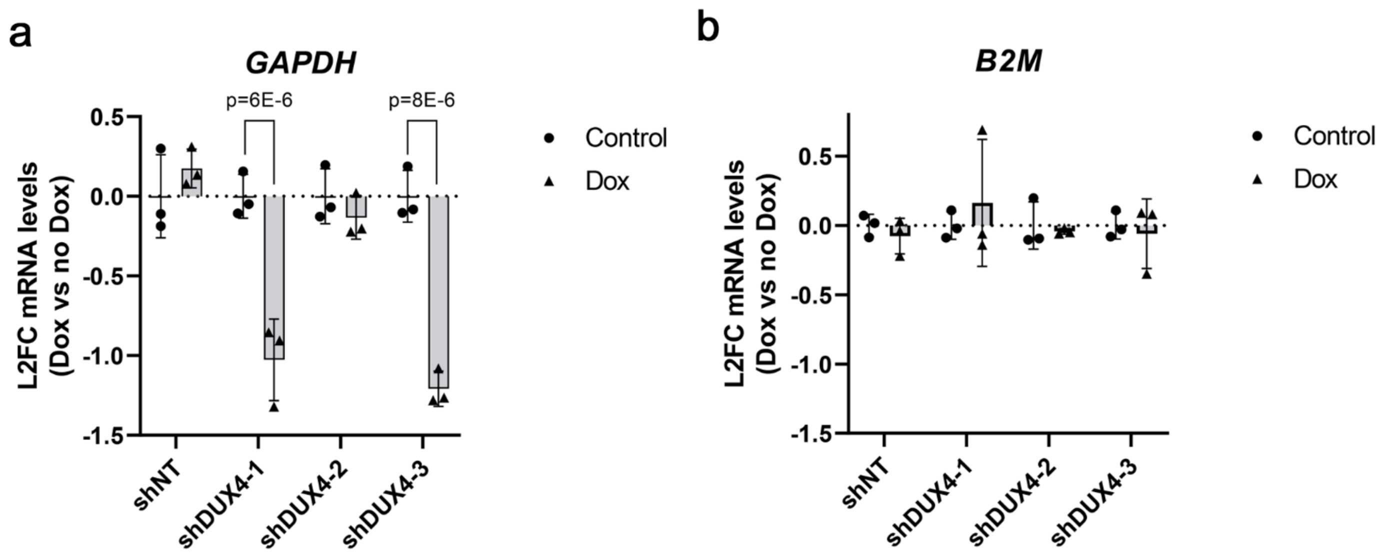

Supplementary Figure S11. **a** and **b**, Log2 fold change of *GAPDH* (a) and *B2M* (b) mRNA levels in CDS-ZH003 cells containing indicated shRNAs after treatment with doxycycline for 4 days to induce shRNA expression. The ΔΔCT method was used for calculation of log2 fold change, with *B2M* as reference gene for *GAPDH* and *ANXA5* as reference gene for *B2M*. n=3 independent experiments, two-way ANOVA, Tukey's multiple comparisons test.

Supplementary Figure S12. ZIP and LOEWE synergy scores for combination treatments of CDS cells with S64315 and chemotherapeutics.

a

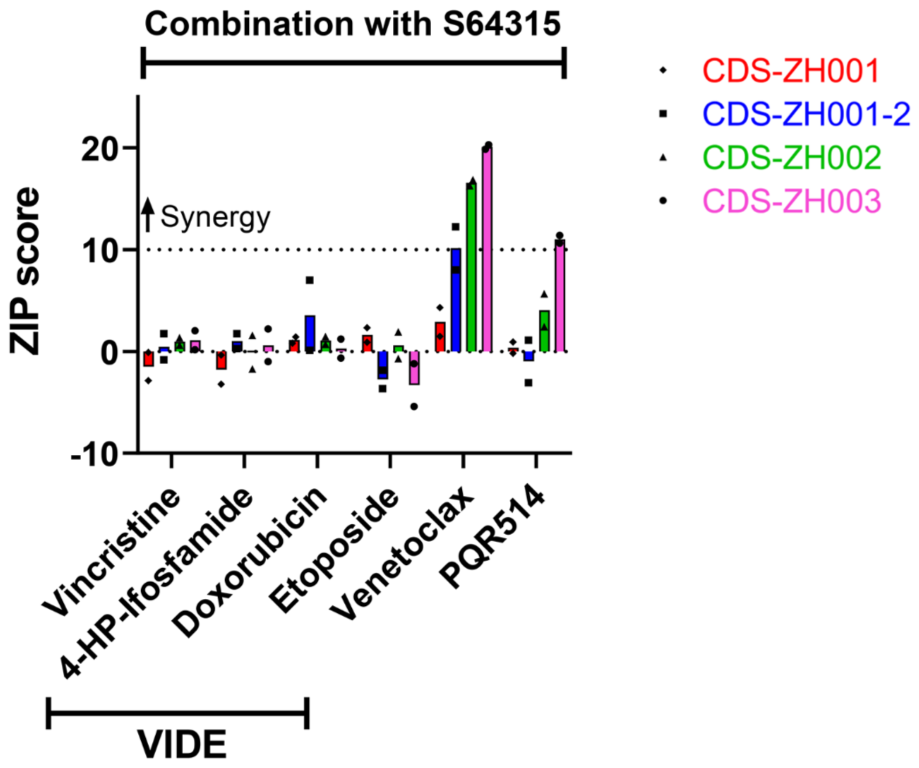

b

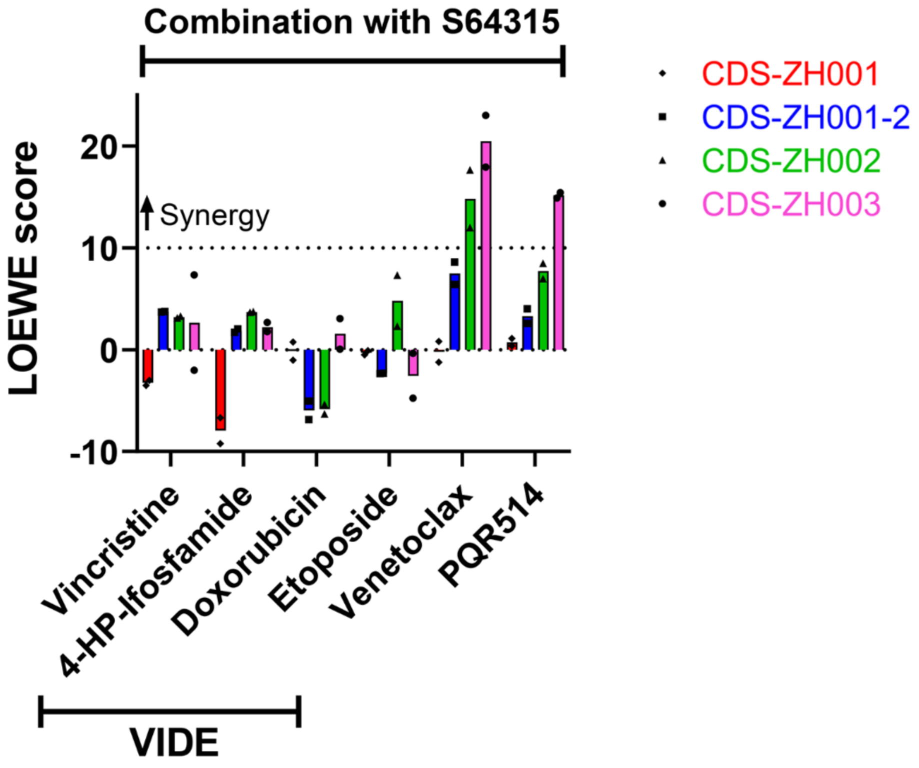

Supplementary Figure S12. **a**, **b** ZIP and LOEWE synergy scores determined for indicated CDS tumoroid models after treatment with S64315 in combination with different chemotherapeutics, venetoclax or PQR514 for 72 h. After treatment, cells were stained with Hoechst 33342 and propidium iodide and dead and live cells were quantified by image-based

analysis using an Operetta high content system. Synergy scores were determined with the number of live cells. (n=2 independent experiments)

Supplementary References

1. Rauh U*, et al.* BRD-810 is a highly selective MCL1 inhibitor with optimized in vivo clearance and robust efficacy in solid and hematological tumor models. *Nat Cancer* **5**, 1479-1493 (2024).

2. Baldauf MC*, et al.* Systematic identification of cancer-specific MHC-binding peptides with RAVEN. *Oncoimmunology* **7**, e1481558 (2018).

3. Bakaric A*, et al.* CIC-DUX4 Chromatin Profiling Reveals New Epigenetic Dependencies and Actionable Therapeutic Targets in CIC-Rearranged Sarcomas. *Cancers (Basel)* **16**, (2024).

Uncropped blots

Blots from Supplementary Figure S6

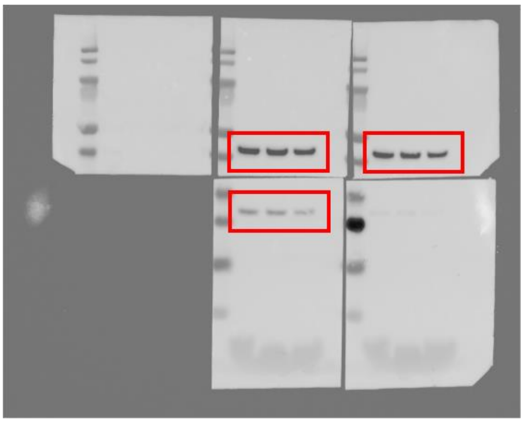

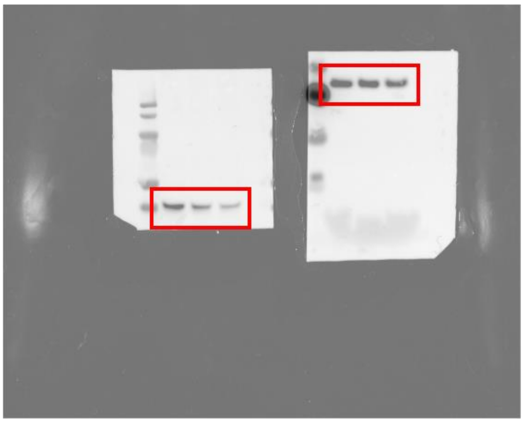

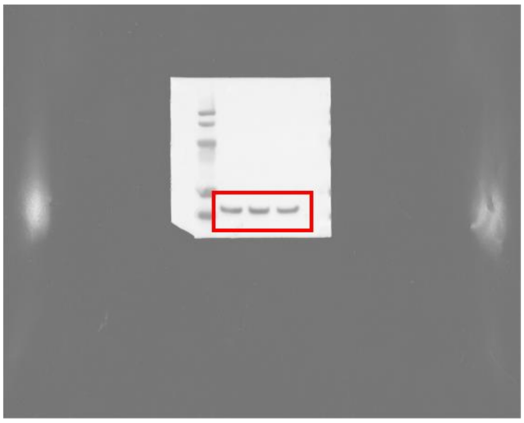

Blots from Supplementary Figure S10

CIC::DUX4 and CIC

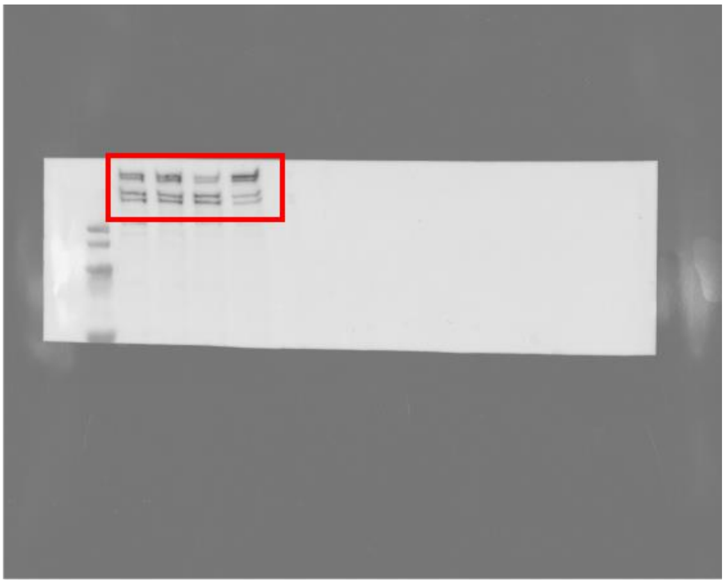

GAPDH

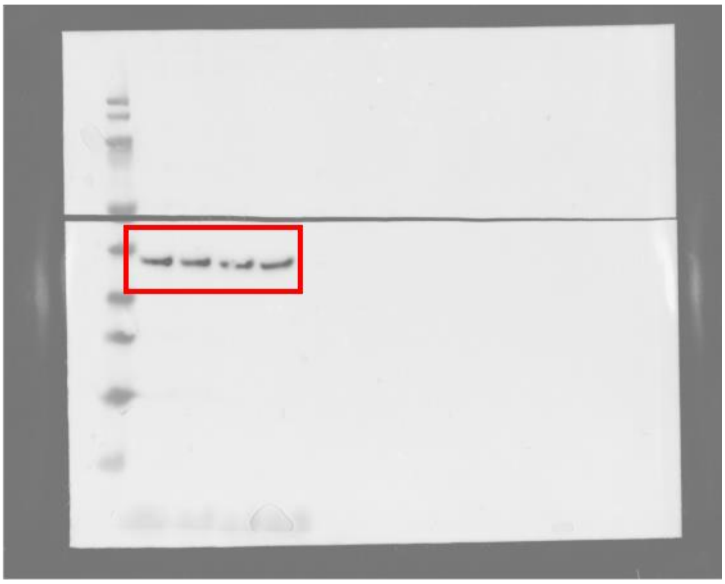

Supplement: Supplementary file 1 — Supplementary Information [file 41467_2025_62629_MOESM1_ESM.pdf]
