# Supplementary material for: Patient-derived tumoroids from CIC::DUX4 rearranged sarcoma identify MCL1 as a therapeutic target
Source: Nat Commun. 2025 Aug 21;16:7688. doi: 10.1038/s41467-025-62629-6 (PMC12370961; doi:10.1038/s41467-025-62629-6)
Supplement: Supplementary file 2 — Description of Additional Supplementary Files [file 41467_2025_62629_MOESM2_ESM.pdf]

Title: Supplementary Data 1

Description: 2000 most variably expressed genes in CDS and EwS tumoroids.

Title: Supplementary Data 2

Description: Genes differentially expressed between CDS and EwS.

Title: Supplementary Data 3

Description: dDSS values
