# Supplementary material for: Patient-derived tumoroids from CIC::DUX4 rearranged sarcoma identify MCL1 as a therapeutic target
Source: Nat Commun. 2025 Aug 21;16:7688. doi: 10.1038/s41467-025-62629-6 (PMC12370961; doi:10.1038/s41467-025-62629-6)
Supplement: Supplementary file 6 — Reporting Summary [file 41467_2025_62629_MOESM6_ESM.pdf]

nature portfolio

Corresponding author(s): Marco Wachtel, Didier Surdez and Beat Schäfer

Last updated by author(s): May 22, 2025

Reporting Summary

Nature Portfolio wishes to improve the reproducibility of the work that we publish. This form provides structure for consistency and transparency in reporting. For further information on Nature Portfolio policies, see our Editorial Policies and the Editorial Policy Checklist.

Statistics

For all statistical analyses, confirm that the following items are present in the figure legend, table legend, main text, or Methods section.

n/a	Confirmed	
☐	☒	The exact sample size (*n*) for each experimental group/condition, given as a discrete number and unit of measurement
☐	☒	A statement on whether measurements were taken from distinct samples or whether the same sample was measured repeatedly
☐	☒	The statistical test(s) used AND whether they are one- or two-sided<br>*Only common tests should be described solely by name; describe more complex techniques in the Methods section.*
☐	☒	A description of all covariates tested
☐	☒	A description of any assumptions or corrections, such as tests of normality and adjustment for multiple comparisons
☐	☒	A full description of the statistical parameters including central tendency (e.g. means) or other basic estimates (e.g. regression coefficient) AND variation (e.g. standard deviation) or associated estimates of uncertainty (e.g. confidence intervals)
☐	☒	For null hypothesis testing, the test statistic (e.g. *F*, *t*, *r*) with confidence intervals, effect sizes, degrees of freedom and *P* value noted<br>*Give P values as exact values whenever suitable.*
☒	☐	For Bayesian analysis, information on the choice of priors and Markov chain Monte Carlo settings
☒	☐	For hierarchical and complex designs, identification of the appropriate level for tests and full reporting of outcomes
☐	☒	Estimates of effect sizes (e.g. Cohen's *d*, Pearson's *r*), indicating how they were calculated

Our web collection on statistics for biologists contains articles on many of the points above.

Software and code

Policy information about availability of computer code

Data collection	RNA-sequencing and whole exom sequencing data collection was performed with Illumina`s NovaSeq 6000 system.<br>DNA methylation was measured with Infinium MethylationEPIC BeadChip Array system.<br>Flow cytometry data collection was performed on an BD LSRFortessa instrument with the BD FACSDiva 8.0.1 software.<br>Luminescence data collection was performed with a BioTek Cytation3 Imaging Reader and the BioTek Gen5 2.07.17 software.<br>qRT-PCR data was collected with on an ABI 7900UT fast real-time PCR system with the SDS 2.4 software.<br>Western blots pictures were taken using a BioRad ChemiDoc Imaging System with the BioRads Image Lab Touch Software 3.0.1.14.<br>Phase contrast pictures were taken with Nikon Eclipse Ts2 and the DS-L4 controler software.
Data analysis	RNA-sequencing and whole exome sequencing data was analyzed on the Galaxy platform (https://usegalaxy.eu/) using the following tools:<br>RNAseq:<br>Trimming of reads: Cutadapt (Galaxy Version 5.0)<br>Alignment of reads to the GRCh38 reference genome: RNA STAR (Galaxy Version 2.7.11a)<br>Counting Read counts per gene: featureCounts (Galaxy Version 2.1.1)<br>Normalization between samples: DESeq2 (Galaxy Version 2.11.40.8)<br>Detection of fusion genes: Arriba (Galaxy Version 2.5.0)<br><br>WES:<br>Trimming and filtering of reads: Trimmomatic (Galaxy Version 0.39)<br>Aliggnment of reads to the hg19 reference genome: BWA-MEM (Galaxy Version 2.2.1)

Filtering reads: Filter BAM (Galaxy Version 2.5.2)
Removal of duplicate reads: RmDup (Galaxy Version 2.0.1)
Homogenization of positional distribution of insertions and deletions in the input using left realignment: BamLeftAlign (Galaxy Version 1.3.9)
Recalibration of read mapping qualities: CalMD (Galaxy Version 2.0.4)
Refiltering of reads based on mapping quality: Filter BAM (Galaxy Version 2.5.2)
Identification of somatic variants: VarScan (Galaxy Version 2.4.2)
Annotation of variants: SnpEff eff (Galaxy Version 5.2)

Methylation analysis:
t-SNE: Rtsne R package (v0.17)

Relative gene expression analysis and gene set enrichment analysis was performed with iDEP1.1 (http://bioinformatics.sdstate.edu/idep11/)
Calculation of classifier scores based on methylation patterns was performed using the sarcoma classifier from the DKFZ (https://www.molecularneuropathology.org/mnp/)
Statistical analysis was performed with Graphpad Prism (v9.3.1) (https://www.graphpad.com/scientific-software/prism/)
Gene tracks with ChIPseq data were visualized with IGV tools (IGV-Web app version 2.0.5) (https://software.broadinstitute.org/software/igv/igvtools)
Flow cytometry data was analyzed with the software FlowJo V10.8.1.
qRT-PCR data was analyzed with the RQ manager 1.2.1 software.
Western blots pictures were processed with the Image Lab 6.1.0 software.
Dose-response curves were generated with Graphpad Prism (v9.3.1) (https://www.graphpad.com/scientific-software/prism/)
Drug synergy was analyzed with the SynergyFinder app (https://synergyfinder.fimm.fi/synergy/20241002100941962 60/)

For manuscripts utilizing custom algorithms or software that are central to the research but not yet described in published literature, software must be made available to editors and reviewers. We strongly encourage code deposition in a community repository (e.g. GitHub). See the Nature Portfolio guidelines for submitting code & software for further information.

Data

Policy information about availability of data

All manuscripts must include a data availability statement. This statement should provide the following information, where applicable:
- Accession codes, unique identifiers, or web links for publicly available datasets
- A description of any restrictions on data availability
- For clinical datasets or third party data, please ensure that the statement adheres to our policy

WES, RNA-seq and DNA methylation data from tumoroid models is deposited in the European Genome-Phenome Archive under the study IDs EGAS50000000379 (WES data; https://ega-archive.org/datasets/EGAD50000000556), EGAS50000000380 (RNA-seq data; https://ega-archive.org/datasets/EGAD50000000557) and EGAS00001008039 (DNA methylation data; https://ega-archive.org/datasets/EGAD00010002760). Previously published ChIP-seq data36 was downloaded from the Gene Expression Omnibus (GEO) database under the accession number GSE248117 (https://www.ncbi.nlm.nih.gov/geo/query/acc.cgi?acc=GSE248117). The remaining data are available within the article, supplementary information or source data file. Source data are provided with this paper.

Research involving human participants, their data, or biological material

Policy information about studies with human participants or human data. See also policy information about sex, gender (identity/presentation), and sexual orientation and race, ethnicity and racism.

Reporting on sex and gender	Patient tumor material for generation of tumoroid models was collected from both male and female patients. Sex of the individual patients ist depicted in supplementary table 1. Our findings apply to both sexes.
Reporting on race, ethnicity, or other socially relevant groupings	No socially constructed or socially relevant categorization was used in the study.
Population characteristics	Patients with Ewing sarcoma included in the study had a mean age of 15 years (range 5-35 years). Patients with CDS included in the study had a mean age of 41 years (range 28-60 years). In all cases diagnosis was confirmed by detection of the corresponding fusion proteins, EWSR1::FLI1 for EwS and CIC::DUX4 for CDS. Samples obtained originated from a wide range of tumor sites and included diagnostic and relapse samples. Furthermore, the cohort included samples from primary and metastatic sites.
Recruitment	All children or adolescents/young adults presenting with Ewing sarcoma or CIC::DUX4 sarcoma at the University Children`s Hospital Zurich or the University Hospital Zurich were included. Additional specimens were obtained from collaborators at the Cantonal Hospital St. Gallen and at the Medical University of Graz. No self-selection process was involved and no other bias is present.
Ethics oversight	Use of the patient material for the experiments was approved by the ethics committee of the Kanton Zurich (BASEC number 2020-01609)

Note that full information on the approval of the study protocol must also be provided in the manuscript.

Field-specific reporting

Life sciences study design

All studies must disclose on these points even when the disclosure is negative.

Sample size	No sample size was pre-determined for ex vivo experiments. Due to the rarity of the tumours under study, all available samples were included. For the drug screening analysis and subsequent in vitro experiments, the number of samples available was sufficient.The number of mice per group for the in vivo studies was estimated using a power analysis and a two-sample, two-sided equality test with the following assumptions: power = 0.8, type I error rate = 1%, untreated group mean tumour volume = 1000 mm^3, treated group mean tumour volume = 600 mm^3, standard deviation = 200 mm^3, sampling ratio = 1. The mean tumour volume values and standard deviation of the groups were estimated based on a pilot experiment.
Data exclusions	No data was excluded.
Replication	Most experiments were performed at least twice, in many cases more than this. All replication attempts were successful and are included.
Randomization	For the xenograft experiment, mice were randomly assigned to individual treatment groups at the beginning of treatment.
Blinding	The investigators were not blinded.

Reporting for specific materials, systems and methods

We require information from authors about some types of materials, experimental systems and methods used in many studies. Here, indicate whether each material, system or method listed is relevant to your study. If you are not sure if a list item applies to your research, read the appropriate section before selecting a response.

Materials & experimental systems

n/a	Involved in the study	
☐	☒	Antibodies
☒	☐	Eukaryotic cell lines
☒	☐	Palaeontology and archaeology
☐	☒	Animals and other organisms
☒	☐	Clinical data
☒	☐	Dual use research of concern
☒	☐	Plants

Methods

n/a	Involved in the study	
☒	☐	ChIP-seq
☐	☒	Flow cytometry
☒	☐	MRI-based neuroimaging

Antibodies

Antibodies used	The following antibodies were used:<br><br>For Western Blot:<br>anti-MCL1 (CellSignaling cat. #4572) Used as 1/1000 dilution.<br>anti-BCL2 (Abcam ab32124) Used as 1/1000 dilution.<br>anti-BCL-XL (CellSignaling cat. #2764) Used as 1/1000 dilution.<br>anti-CIC (Novus Biologicals #NB110-59905SS) Used as 1/1000 dilution.<br>anti-PARP (CellSignaling cat. #) Used as 1/1000 dilution.<br>anti-GAPDH (CellSignaling cat. #2118S) Used as 1/1000 dilution.<br>anti-b-Actin (CellSignaling cat. #4970) Used as 1/1000 dilution.<br>anti-rabbit IgG-HRP (Cell Signaling Technologies cat. #7074). Used as 1/2000 dilution.
Validation	anti-MCL1 (CellSignaling cat. #4572): Validation by Cell Signaling Technologies: Was shown to detect endogenous levels of human MCL1 protein. (https://www.cellsignal.com/products/primary-antibodies/mcl-1-antibody/4572?srsltid=AfmBOoot8WRf_1aud41Xhz21p1eS5rSg6NkfjWM4v-L9aM1V4ed_tS_v).<br><br>anti-BCL2 (Abcam ab32124): Validation by Abcam: Was shown to detect endogenous levels of human BCL2 protein. (https://www.abcam.com/en-us/products/primary-antibodies/bcl-2-antibody-e17-ab32124#application=wb).<br><br>anti-BCL-XL (CellSignaling cat. #2764): Validation by Cell Signaling Technologies: Was shown to detect endogenous levels of human BCL-XL protein. (https://www.cellsignal.com/products/primary-antibodies/bcl-xl-54h6-rabbit-mab/2764?srsltid=AfmBOoqffB6nhoiS-vAJiWx6nuwoXCxlly_ZTk2AB_P9DK_3qeZaDUmL).<br><br>anti-CIC (Novus Biologicals #NB110-59905SS): Validation by Novus Biologicals: Was shown to detect immunoprecipitated CIC from HeLa lysates (https://www.novusbio.com/products/capicua-antibody_nb110-59905#reviews-publications).<br><br>anti-PARP (CellSignaling #): Validation by Cell Signaling Technologies: Was shown to detect endogenous levels of uncleaved and

cleaved human PARP protein (https://www.cellsignal.com/products/primary-antibodies/parp-antibody/9542?srsltid=AfmBOoqFLbT9sYL-T9ygsOgRy2i3rtrRTe9gpBhTasET-ReLbDQRPBCy).

anti-GAPDH (CellSignaling cat. #2118S): Validation by Cell Signaling Technologies: Was shown to detect endogenous levels of human GAPDH protein. (https://www.cellsignal.com/products/primary-antibodies/gapdh-14c10-rabbit-mab/2118).

anti b-Actin (CellSignaling cat. #4970): Validation by Cell Signaling Technologies: Was shown to detect endogenous levels of human b-Actin protein. (https://www.cellsignal.com/products/primary-antibodies/b-actin-13e5-rabbit-mab/4970).

Animals and other research organisms

Policy information about studies involving animals; ARRIVE guidelines recommended for reporting animal research, and Sex and Gender in Research

Laboratory animals	Female NSG mice (NOD.Cg-Prkdcscid Il2rgtm1Wjl/SzJ, Charles River F, L'Arbresle, France) aged between 7 and 10 weeks were used for the xenograft experiments.
Wild animals	The study did not involve wild animals.
Reporting on sex	The xenograft experiment was performed with female mice only.
Field-collected samples	The study did not involve samples collected from the field.
Ethics oversight	The animal experiments have been approved by the veterinary service of the canton of Zurich and were performed according to the animal license ZH013/2021.

Note that full information on the approval of the study protocol must also be provided in the manuscript.

Plants

Seed stocks	*Report on the source of all seed stocks or other plant material used. If applicable, state the seed stock centre and catalogue number. If plant specimens were collected from the field, describe the collection location, date and sampling procedures.*
Novel plant genotypes	*Describe the methods by which all novel plant genotypes were produced. This includes those generated by transgenic approaches, gene editing, chemical/radiation-based mutagenesis and hybridization. For transgenic lines, describe the transformation method, the number of independent lines analyzed and the generation upon which experiments were performed. For gene-edited lines, describe the editor used, the endogenous sequence targeted for editing, the targeting guide RNA sequence (if applicable) and how the editor was applied.*
Authentication	*Describe any authentication procedures for each seed stock used or novel genotype generated. Describe any experiments used to assess the effect of a mutation and, where applicable, how potential secondary effects (e.g. second site T-DNA insertions, mosiacism, off-target gene editing) were examined.*

Flow Cytometry

Plots

Confirm that:

- [x] The axis labels state the marker and fluorochrome used (e.g. CD4-FITC).
- [x] The axis scales are clearly visible. Include numbers along axes only for bottom left plot of group (a 'group' is an analysis of identical markers).
- [x] All plots are contour plots with outliers or pseudocolor plots.
- [x] A numerical value for number of cells or percentage (with statistics) is provided.

Methodology

Sample preparation	For competition assays:<br>Cells were first transduced with mCherry-labelled Lenticrispr sgRNA constructs. 3 days after transduction, cells were mixed with untransduced cells as a reference in a 1:1 ratio and the fraction of mCherry-positive to mCherry-negative cells was quantified by flow cytometry on a Fortessa flow cytometer (BD Biosciences). 7 days later, the fraction was again determined and the ratio of mCherry-positive fractions between day 2 and day 7 was calculated. Before flow cytometric analysis, cells were detached from dishes, fixed with 0.5 % PFA for 5 min and washed with PBS.<br><br>For cell death assays:<br>Cells were grown and treated in 6-well plates. After treatment, remaining adherent cells were detached with Accutase (Sigma-Aldrich, A6964) (1:2 diluted with PBS) and combined with floating cells in a centrifuge tube. After washing twice with PBS, single cells were resuspended in 100 microl Annexin V binding buffer (BD Biosciences, #556454) and stained with 5 microl Annexin V-APC (BD Biosciences, #550474) and 5 microl 7-AAD (BD Biosciences #559925) for 15 min at room temperature in the dark. After dilution with additional 400 microl binding buffer, cells were filtered through a cell strainer

	into a FACS tube and analyzed.
Instrument	BD LSRFortessa Cell Analyzer
Software	For data collection, the BD FACSDiva 8.0.1 software was used. For data analysis the software FlowJo_V10.8.1 was used.
Cell population abundance	Competition assay:<br>Cells were 50-80% mCherry positive after transduction with the sgRNA plasmid and therefore accounted for about 25-40% after mixing them in a 1:1 ratio.<br><br>Cell death assay:<br>Between 10% and 50% of the cells were double positive for Annexin V and 7-AAD after treatment with MCL1 inhibitor.
Gating strategy	Competition assay:<br>First, viable cells were gated based on the FSC area /SSC area signals. Then singlet cells were gated based on the FCS area/FCS height signals. Transduced cells expressing mCherry fluorescent protein were selected based on fluorescence channel with the help of an untransduced control.<br><br>Cell death assay:<br>First, very small debris was exluded based on the FSC area /SSC area signals. Then singlet cells were gated based on FCS area/ FCS height signals. Signals from Annexin V and 7-AAD were gated based on an untreated control.<br>Compensation was performed with unstained and single stained controls.

☐ Tick this box to confirm that a figure exemplifying the gating strategy is provided in the Supplementary Information.
